# Ecologies of Resilience for Australian High School Students from Refugee Backgrounds: Quantitative Study

**DOI:** 10.3390/ijerph19020748

**Published:** 2022-01-10

**Authors:** Emily Miller, Tahereh Ziaian, Helena de Anstiss, Melanie Baak

**Affiliations:** 1 Justice and Society, University of South Australia, Adelaide 5001, Australia; tahereh.ziaian@unisa.edu.au (T.Z.); Helena.deanstiss@unisa.edu.au (H.d.A.); 2 Education Futures, University of South Australia, Adelaide 5001, Australia; melanie.baak@unisa.edu.au

**Keywords:** refugee, student, school, resilience, resettlement, mental health, wellbeing

## Abstract

Forced displacement of refugees, currently at record levels, leads to increased cultural diversity in many countries with benefits and challenges for individuals, communities, and societies. Refugees often face significant stressors both pre- and post-migration, and hence are at increased risk of poor mental health and wellbeing. Children and adolescents make up a significant proportion of refugees globally, and hence mental health supports for these young people are crucial. Current mental health research often uses pathologized approaches that focus on trauma, although there is growing literature highlighting the importance of a sense of belonging and the reduction in discrimination and social exclusion, emphasizing strengths and agency of individuals and communities. Resilience is often noted for its positive influence on mental health and wellbeing; however, research regarding how mechanisms of resilience function is still developing. This study investigated mental health and wellbeing of refugee-background Australian youth to better understand the role and function of resilience. Findings suggest that intersecting social ecologies, such as those within family, school, or community networks, contribute to development of identity and a sense of belonging for youth, which together form a resilient system that provides resources for wellbeing. Adaptations of school policy and practice can support positive mental health and wellbeing outcomes by contributing to and developing resilient environments, such as through building connections to family, improving positive recognition of cultural identity for individuals and across the whole school community, and actively working to minimize discrimination.

## 1. Introduction

Global migration, either temporary, permanent, voluntary or forced, contributes to growing cultural diversity in many societies. Of note, involuntary migration, or forcible displacement, is currently at record levels [1], with potential for continued growth as a result of people’s exposure to violence, economic challenges, or climate change [2]. Children and young people make up over 42% of forcibly displaced peoples, compared to only 30% of the total global population [3], some of whom go on to be formally recognized as refugees. As such, the educational, developmental, and mental health needs of refugee children and youth are a crucial issue for research.

Government policies and programs which aim to address the challenges experienced by refugees and asylum seekers vary; however, one durable solution for refugees is resettlement (explained in detail below). The Australian Government offers refugee resettlement opportunities to an annual quota of refugees [4], adding to the cultural diversity of an already multicultural country in which nearly half the population were either born overseas or have one parent who was born overseas [5]. Australia therefore provides a setting within which youth resettlement can be investigated, to both understand the needs of refugee background youth and to provide evidence that contributes to the development of specific policies and practices that support their mental health and wellbeing. This study investigates how mental health and wellbeing of refugee background youth can be promoted through a resilience lens. As described in detail below, we take resilience to be features within social systems or structures, such as in school or in families and community groups, that enable positive outcomes for individuals after adversity.

A focus on positive aspects of agency and resilience can provide scope for policy and practice in a range of settings that identifies and builds upon refugees’ strengths and capacities as they face challenges; however, in global refugee policy the term resilience can remain linked by comparison to individualized notions of who is not resilient, and therefore vulnerable or at-risk [6]. In the work presented here, the influence of multiple social ecologies that develop within school contexts, family groups, or within communities, is highlighted in the hope of contributing to discourse that considers resilience as an element of complex systems which enable access to resources. As the concept of resilience pervades policy in governments and institutions, it is important to identify how resilient ecologies function, rather than placing a spotlight on the individuals for whom these ecologies provide support [7].

The study presented here is the quantitative component of a mixed-methods study investigating experiences of refugee background youth studying in Australian high schools. Analysis of survey data examines the mediating role of resilience in mental health and wellbeing outcomes. These processes are discussed in terms of how school practice can connect with and contribute to resilient ecologies to support the mental health and wellbeing of young people from refugee backgrounds.

### 1.1. Refugee Resettlement

Although most forcibly displaced peoples live in or near their country of origin [1], there are opportunities for some of those officially recognized as refugees by the United Nations to access resettlement, which entails relocation from a country of asylum to a third country where they are subsequently provided with the legal right to permanent residency, with pathways to citizenship [8]. Resettlement supports depend on settlement location in terms of type and duration, with an overall goal of positive integration outcomes, which rely on a combination of effective policies and implementation, and understanding of settlement dynamics [9,10]. For youth who may have experienced considerable obstacles to accessing education during their refugee experience, engagement with education in resettlement can pose challenges but also offers hope for positive settlement and possibilities for the future [11].

Contemporary research uses various theoretical approaches to identify and understand complexities of how people settle in new places, particularly within culturally diverse societies such as Australia [12]. Although debate regarding theories of cultural contact or change abound, there is a general convergence towards a notion that individuals’ cultural practices and norms can undergo a process of change when two or more groups interact [13], that both newcomers and members of a settlement society may make adaptations that vary depending on context [14], and that the process for newcomers relies on a range of factors such as access to services, education, employment, and social networks [15,16]. Recent scholarship attends to how multiple and complex social relationships also affect settlement, relationships which may extend within cultural or family groups, or national, diasporic, or transnational contexts [17]. Resilience has been noted as a key facilitator of positive outcomes such as mental health and wellbeing for people from refugee backgrounds in resettlement [10].

In the study presented here, we focus on this process of settlement for youth from refugee backgrounds in Australia. More specifically, we outline some of the contextual environments, or ecologies (as described further below), that facilitate mental health and wellbeing for youth. Here we focus specifically on the experiences of refugee youth, as their unique experiences warrant focused research [18]. In the following section we outline the resilience literature and how it relates to youth from refugee backgrounds.

### 1.2. The Resilience Literature and Ecologies of Resilience

Resilience as a construct has been theorized as comprising two parts, namely that stressful or challenging experiences have occurred and that responses to these stressors can ultimately lead to positive mental health and wellbeing outcomes [19,20]. For youth from refugee backgrounds, the first part of the resilience construct can be assumed to have occurred, namely challenges, traumas or stressors, albeit on a spectrum as refugee experiences are highly heterogenous. With regard to the second element of the construct, studies of resilience consistently identify positive processes and outcomes for former refugees, particularly youth, in relation to social support, and a sense of connection and belonging [21,22,23,24,25,26], often with a specific focus on the contextual settings that contribute to resilience [27,28,29,30,31].

In earlier psychological research into challenging experiences such as trauma, and people’s responses to these challenges, there was a focus on pathology and negative human adaptations under stress; however, resilience research now instead focuses on processes that foster and promote positive outcomes such as health and wellbeing [19]. Although there has been this trend towards a focus on strengths, and growth in research investigating predictors of resilience or protective factors, there remains little common ground across disciplines or studies about how the concept is operationalized [20], an issue made more complex when considering cross-cultural contexts [32].

Ungar and colleagues [33] developed a conceptual framework regarding resilience using a social-ecological theory that draws on the foundational work of Bronfenbrenner and the ecological systems theory [34]. Bronfenbrenner’s systems are diagrammed as a set of nested circles that represent different layers of social ecologies—namely macro-, exo-, meso-, micro- and chrono-systems—which can influence youth development such as broad societal cultural norms and laws (macro-system), or close personal relationships within families (micro-system), for example [34].

Ungar and colleagues have suggested that the ecological systems theory provides a heuristic that can be employed to identify different aspects of a young person’s environment that relate to resilience, with three guiding principles relating to the ecological systems to consider, namely those of equifinality, differential impact, and cultural moderation [33]. Equifinality draws on evidence that suggests all elements of the micro-, meso-, exo-, macro-, and chrono- systems are equally able to have significant impact on youth resilience. Differential impact notes that there are different ways in which actions or processes impact individuals depending on circumstance; there is no singular way in which resilience is constructed for all people. Cultural moderation is concerned with noting that there are expressions and experiences of resilience that are different across cultural settings; resilience can differ in its mechanisms across cultural groups where the environmental factors that provide access to relevant resources are key [35].

The social-ecological theory notes that there are two-way interactions between a person and their environment that continuously evolve, providing access (or not) to supports and resources for resilience [33] and that a focus on context is needed to understand mechanisms of resilience rather than a focus on the individual [36]. Ungar and colleagues further discuss how understanding and experiences of resilience depend on the ways in which several tensions are able to be resolved regarding: access to material resources; relationships; identity; power and control; cultural adherence; social justice; and, cohesion [35]. This focus on contextual issues is a critical step towards recognition that responses to traumatic events or life stressors are normal human reactions rather than necessarily maladaptive behaviors, and that building resilient environments that support refugee background youth can be crucial to supporting positive mental health and wellbeing outcomes [37].

We use a definition of resilience as Ungar has suggested and consider resilience to be both “an individual’s capacity to navigate to health resources and a condition of the individual’s family, community and culture to provide these resources in culturally meaningful ways” [38]. In this study, we investigate the “conditions” that we identify as ecologies of resilience, drawing on the notion that there are multiple overlaid and intersecting social ecologies that affect individual youth as described by Bronfenbrenner [34] and elaborated by Ungar and colleagues [33]. This is akin to theorization of “social resilience”, which considers people’s resilience as the capability to face and cope with stressors through access to economic, social, and cultural resources which are “embodied in networks, social hierarchies, and cultural repertoires” [39] (p. 14). The study presented here specifically investigates how the overlapping ecologies that are created by family and community networks of support, both at school and in other contexts, can provide access to a sense of belonging and identity, and emotional or practical resources for youth to support wellbeing.

### 1.3. Challenges and Resources for Refugee Background Youth Mental Health and Wellbeing

This study was conducted to address the mental health and wellbeing of youth from refugee backgrounds as their heterogenous experiences can place them at increased risk of negative outcomes. Youth with refugee backgrounds may have had challenging or traumatic pre-migration experiences that directly impact their own mental health and wellbeing, and they may also be negotiating the effects of traumatic experiences on other family members [40]. In addition, postmigration stressors and challenges have been noted as crucial factors impacting wellbeing [41,42]. Some youth may arrive in a resettlement country without family and must draw on alternative resources to adjust in their new home [43]. Youth who settle with family may face intergenerational issues and challenging family dynamics in resettlement which are complex, providing challenges in addition to protective factors [44,45]. Youth may have had interrupted or missing periods of education pre-migration that can impact engagement with education in resettlement [46,47], particularly for pre-literate learners working towards majority language proficiency [48]. Experiences of belonging may be elusive, particularly in the presence of discrimination or racism [49,50,51].

Despite these challenges and difficulties for refugee youth in resettlement, the literature also highlights positive processes such as familial or community supports or school practices that support wellbeing. Whole school approaches to engendering belonging for all students have been noted as a positive process [52,53], as has connection to family supports and connections with cultural community [28,29,54]. Identity development is a critical element of adolescence and, as such, is also a crucial factor to consider for refugee youth who navigate this identity development in a context of resettlement processes [55]. It has been noted that connections to heritage culture and with the majority culture of a settlement country enable positive mental health outcomes in terms of youth identity development in settlement contexts [56].

The resilience construct has consistently been used to encapsulate the positive outcomes after adversity that resettled refugee youth can exemplify [22]; however, the term can be susceptible to a focus on the individual rather than their environment. This not only poses a risk of reinforcing a dichotomy of vulnerable versus resilient, but also places responsibility for outcomes on individuals rather than on the systems that they navigate [6]. In this study, we turn the lens towards the development of resilient ecologies that provide resources for mental health and wellbeing of youth from refugee backgrounds.

In the next section we describe the study conducted during 2017–2019 with youth from refugee backgrounds resettled in Australia. The study aimed to investigate how resilient ecologies operate to support positive mental health and wellbeing outcomes. We sought to answer a research question that asked how school practice contributes to resilient ecologies that support mental health and wellbeing of youth from refugee backgrounds. Drawing on the extant research, we proposed three hypotheses regarding the impact of discrimination, identity development, and school and family support on mental health and wellbeing. We draw on research scales that consider identity development in terms of cultural identity, i.e., ethnic and national cultures, as these have been noted as central elements of identity for resettling youth from refugee backgrounds [55,56]. The hypotheses represent predictions that these factors have a direct effect on mental health and wellbeing, and that resilience also mediates these effects.

### 1.4. Hypotheses

**Hypothesis** **1** **(H1).** *Discrimination reduces life satisfaction and increases psychological distress.*

**Hypothesis** **2** **(H2).** *Ethnic identity, national identity, family congruence, and school support all increase life satisfaction and reduce psychological distress.*

**Hypothesis** **3** **(H3).** *Resilience mediates effects of discrimination, ethnic identity, national identity, family congruence, and school support on life satisfaction and psychological distress.*

## 2. Materials and Methods

This article presents analysis of quantitative data collected via a survey questionnaire with 322 high school students from refugee backgrounds in Australia. The study was conducted using a subset of quantitative data from a larger mixed methods project. This mixed methods project was conducted in South Australia and obtained ethical approvals from the University of South Australia and relevant education sectors. The larger project data and analysis are not presented here.

The survey was created by a team of researchers and contained questions relating to several sociodemographic variables and scales as described in the following sections. Data were collected collaboratively with support from the research team, industry partners, and bilingual workers. Details of this research project and the collaborative data collection process can be found in other publications [57,58]. In brief, data were collected in South Australia throughout 2017–2018: youth participants were presented with a survey in a paper format and were supported through informed consent processes by bilingual workers and/or interpreters where relevant. The bilingual workers also provided cultural or linguistic assistance to the participating youth by remaining close-by to answer any queries about the questions during survey completion, or to assist in any other way by directing them to other services following participation.

### 2.1. Participants

Participants were youth from refugee backgrounds aged between 14 and 26 years. All youth in this study were studying in the final years of high school at the time of survey completion. The study aimed to invite participants from three major world regions of Africa, the Middle East, and South Asia, which was representative of refugee migration patterns in the years prior to study inception [59]. All youth were in contact with family, which was taken to be inclusive of either living with or being in contact with parents or with extended family, siblings, or other caregivers, rather than only the nuclear family unit. Youth had been in Australia for between one and 15 years. See Table 1 for more detail. Criteria for inclusion in the study required participants to be legally granted the right to live permanently in Australia (i.e., no participants had temporary visa status), and to have migrated after refugee experiences. They were not the children of migrants but had themselves travelled to Australia as children, adolescents, or young adults. Hence, participants were from a “refugee background” as distinct from a more general migrant background.

The experiences of youth with refugee backgrounds in Australia with temporary visa status (i.e., who are seeking asylum) are beyond the scope of the work presented here, particularly as temporary visa status has a significant effect on mental health outcomes [60], which are the focus of this study.

### 2.2. Measures

Socio-demographic details were determined from direct questions regarding gender (male/female options only), age, time in Australia, and regional background (determined by responses to a question asking about ethnic or cultural background, with data then collated into regions of Africa/Middle East/South Asia). Several scale variables were produced, detailed below (see also Table 1).

#### 2.2.1. Ethnic and National Identity

Assessment of identity in terms of participants’ affinity with their ethnic or cultural background and/or the culture of majority Australian society was undertaken via questions drawn from the Mutual Intercultural Relations in Plural Societies (MIRIPS) study, which has been conducted across a wide range of contexts and with diverse participant cohorts [61]. In this study, five items regarding ethnic identity were presented to participants with Likert responses ranging from 1 = strongly disagree to 5 = Strongly agree, such as “Being part of and participating in my ethnic culture makes me feel happy”, with Cronbach’s alpha (α) = 0.892. Three items regarding national identity were presented with the same response range, such as “I feel that I am part of the Australian culture (the culture of majority/broader society)”, with α = 0.814. For each of these scales, data were recoded using the mean of responses to form single variables for analysis, with a higher score indicating a higher level of identification with either construct.

#### 2.2.2. Family Congruence

Family congruence items assessed how youth perceived that they and their family members were in alignment in terms of lifestyle and values. Seven items were drawn from the Intergenerational Congruence in Immigrant Families-Child Scale [62], which has been used with a range of diverse groups including people from refugee backgrounds in resettlement contexts [e.g., Betancourt and colleagues [63]]. In this study, seven statements were presented with Likert responses ranging from 1 = strongly disagree to 5 = Strongly agree, such as “My family and I agree on the aims, goals and things believed to be important in life”, with α = 0.901. Data were recoded using the mean of responses, with a higher score indicating higher congruence.

#### 2.2.3. Discrimination

Perceptions of discrimination were assessed using five items drawn from the MIRIPS study (detailed above) [61]. The items in this study focused on youth experiences of discrimination towards their cultural group from majority Australian society. Items had a Likert response range from 1 = strongly disagree to 5 = Strongly agree for statements such as “I have been teased or insulted because of my cultural background”, with α = 0.804. Data were recoded using mean of responses, with a higher score indicating higher levels of discrimination.

#### 2.2.4. Resilience

Resilience was assessed using ten items from the Conner–Davidson Resilience Scale, which assessed participants’ perceptions of how they respond to adversity on a personal level [64]. An abridged example item is, “I am not easily discouraged…by failure”. Participants were asked to indicate the extent of how true ten statements like this were over the past one month, with a response range from 1 = Not at all true to 5 = True nearly all of the time, with α = 0. 855. Data were recoded using mean of responses with a higher score indicating higher resilience.

#### 2.2.5. School Support

Participants’ perceptions of how they felt they belonged and were supported at school were assessed using adapted elements of the National Schools Opinion Survey [65], the Trends in International Maths and Science Study [66], and the Programme for International Student Assessment [67] studies. Seven items were presented as statements with a response range from 1 = Always to 4 = Never, such as “My teachers notice when I am doing a good job and let me know about it” and “I feel safe at my school”, with α = 0.856. Data were recoded via the mean of items, with a higher score indicating a greater sense of belonging and support at school.

#### 2.2.6. Mental Health and Wellbeing

Two scales were used to assess mental health and wellbeing in terms of both life satisfaction and psychological distress.

Life satisfaction was assessed using the Satisfaction with Life Scale [68,69]. Five items presented a statement such as “So far, I have got the things I want in life” with a Likert response set ranging from 1 = Strongly disagree to 5 = Strongly agree, with α = 0.775. Data were recoded using mean of the item scores, with a higher score indicating higher satisfaction with life.

Psychological distress was assessed using ten items asking about how participants felt over the past four weeks, using the Kessler Ten, or K-10 [70,71]. Statements such as “In the past four weeks how often did you feel tired out for no good reason?” were posed and responses were in a Likert format ranging from 1 = None of the time to 5 = All of the time, with α = 0.891. Data were recoded using the sum of responses, with a higher score indicating higher levels of psychological distress.

### 2.3. Data Treatment and Analysis

Bivariate correlation analyses, ANOVAs, *t*-tests and assumption tests for regressions were conducted using IBM SPSS Statistics software (Version 26.0) (Adelaide, Australia). Structural equation modelling (SEM) was conducted using AMOS software (Version 26.0). The component score of each scale was used in the path model. Eighty percent of individual items (i.e., not scale scores, rather each individual item) had less than 2% missing (and overall highest missing = 4.6%), and hence were eligible for imputation [72]. Missing data were imputed using the mean of other responses on individual scales for each participant. Missing data for the length of time in Australia were imputed using series mean for the SEM only (three cases missing), with no missing data on other socio-demographic variables.

## 3. Results

In total, 322 youth participants’ data were included in this study. Around ~28% had been in Australia for less than two years, ~31% for two to five years, ~28% between five and ten years, and ~12% more than ten years. Around one-third identified as male (*n* = 116, 36%) and two-thirds female (*n* = 206, 64%). The majority of participants had backgrounds in countries located in the region of the Middle East (*n* = 133, ~41%), followed by the African continent (*n* = 102, ~32%) and Southern Asia (*n* = 87, 27%), reflecting recent refugee youth migration patterns [59]. Participants were mostly aged 15–25 years; however, six youth were just about to turn 15 years and one had only just turned 26 years old. Most participants (~87%) were aged 20 years or younger, reflecting the general trend in Australia that students complete high school during their teenage years. Some participants were slightly older, which can be more usual for youth who arrive in Australia at an older age and work to complete Australian schooling qualifications whilst also learning a new language (for many) and learning to navigate new systems in settlement.

As displayed in Table 1, in general, participants reported moderate to high levels of satisfaction with life, feelings of resilience, and identification with what they perceived to be the national settlement culture of Australia, and with their self-identified ethnic or cultural group. Most reported generally positive experiences of school support, in addition to feeling aligned with their family members in terms of values and lifestyle choices.

Correlations between discrimination and all other scale variables were significant. Youth who reported higher levels of discrimination were more likely to report lower levels of identification with either national or ethnic groups, lower sense of congruence within their families, and poorer mental health and wellbeing outcomes. As discrimination due to visual markers such as skin color or dress have been noted to be linked to discrimination [73], a one way between-subject analysis of variance (ANOVA) was conducted. Region of origin was not a significant factor related to experiences of discrimination [*F*(2,319) = 0.658, *p* = 0.519] when comparing youth with backgrounds in three regions of Africa, the Middle East, or South Asia.

Gender significantly correlated with satisfaction with life and resilience. Further analysis via *t*-tests showed that females had significantly lower life satisfaction (*M* = 3.45, *SD* = 0.75) compared to males (*M* = 3.68, *SD* = 0.80); *t*(320) = 2.57, *p* = 0.011. Females also reported lower resilience (*M* = 3.49, *SD* = 0.68) compared to males (*M* = 3.79, *SD* = 0.73); *t*(320) = 3.74, *p* < 0.001. To investigate this further, the data were split into three groups according to region of origin and *t*-tests were conducted again, this time showing that gendered differences within regional groups was non-significant for those from African backgrounds or Asian backgrounds, but was significant for those with backgrounds in the Middle East. Middle Eastern females reported lower resilience (*M* = 3.38, *SD* = 0.65) compared to males (*M* = 3.92, *SD* = 0.67); *t*(131) = 4.62, *p* < 0.001, and lower life satisfaction (*M* = 3.44, *SD* = 0.75) compared to males (*M* = 3.71, *SD* = 0.76); *t*(131) = 2.04, *p* = 0.043.

Time in Australia also showed significant correlations; the longer youth had been living in Australia, the more likely they were to report experiences of discrimination, lower levels of identification with the national society, lower levels of feeling supported at school, and lower levels of family congruence. ANOVA testing revealed that there were significant regional disparities in the length of time spent in Australia [*F*(2,19.81, *p* < 0.001], with a significantly lower mean for those with Middle Eastern backgrounds (*M* = 3.83, *SD* = 3.03) compared to the other two regions of Africa (*M* = 6.62, *SD* = 4.30) and South Asia (*M* = 5.54, *SD* = 2.74), and no significant difference in length of time in Australia between Africa and South Asia.

**Figure 1:**
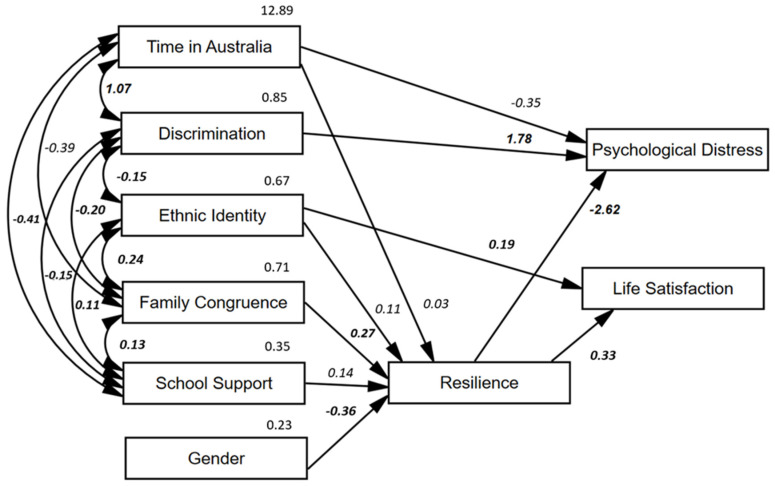
Mediation path model, with significant correlations and effects displayed (bold *p* < 0.001/not-bold *p* < 0.05.

Further analysis considered relationships between variables using SEM. In the initial path model, age, gender, time in Australia, discrimination, ethnic identity, national identity, family congruence, and school support were inputted as covarying independent variables and resilience as a mediator variable. Pathways were drawn to test for mediation by resilience of all predictor variables, in addition to direct effects on the outcomes of life satisfaction and psychological distress. All non-significant pathways were removed to preserve parsimony and, hence, national identity and age were removed due to the lack of significant effects. The final model is displayed in Figure 1. The model showed acceptable fit indices as shown in Table 2 (Model 1). Model invariance was tested using three groups, namely region of origin (Africa/Middle East/South Asia), and fit indices were acceptable as shown in Table 2 (Model 2).

As shown in the mediation path model in Figure 1, length of time in Australia and gender both had a significant effect on outcome variables that were mediated by resilience, and time in Australia also had a direct effect on psychological distress. Gender was not significantly correlated with any exogenous variables. However, longer time in Australia correlated with higher experiences of discrimination, lower levels of family congruence, and lower perceptions of school support.

Tests of direct effects and mediation by resilience were considered for all exogenous variables (gender, time in Australia, discrimination, family congruence, ethnic identity, school support) on the two outcome variables of life satisfaction and psychological distress. Discrimination was not mediated by resilience but did have a direct effect on psychological distress. Family congruence and school support were fully mediated by resilience. Ethnic identity was mediated by resilience and had a direct effect on life satisfaction. Significant direct and mediated effects of control and scale variables are shown in Table 3.

A multigroup analysis was conducted with individual structural path weights constrained to be equal to determine significant difference between effects dependent on region of origin. The only path when constrained that caused significantly different model comparison (*χ*2(2) = 6.059, *p* = 0.048) was from gender to resilience. The effect of resilience on life satisfaction or psychological distress was not significantly different between groups, indicating that although males and females with different regional backgrounds expressed significantly different within-region levels of resilience, the impact of this resilience on outcome variables remained significant regardless of gender.

To check further for multigroup differences for all direct and indirect effects, previously withdrawn non-significant paths were re-tested for significance across regional groups, with the only significantly different outcome noted for the pathways from Family Congruence to Psychological distress, with model comparison *p* = 0.010. This path model had an acceptable fit, as shown by results for Model 3 in Table 2. The effect of family congruence on psychological distress was non-significant for participants from the Middle East and Africa, but significant for those with a background in South Asia; *β* (Standardized) = −0.293, *p* = 0.004 (CI−0.489, −0.092).

Hypotheses were only partially supported. H1 was found to be true for the direct significant effect of discrimination on psychological distress; however, the direct effect of discrimination on life satisfaction was non-significant. H2 was partially supported with a significant direct effect of ethnic identity on life satisfaction; however, there were no other significant effects of the four variables on either life satisfaction or psychological distress in the model. H3 was found to be supported for significant mediation by resilience of ethnic identity, family congruence, and school support; however, there was no mediation of either discrimination or national identity. H2 and H3 proposed that national identity would play a significant role in the model; however, this was not found to be the case.

## 4. Discussion

In this study we considered a range of factors that impact youth resilience and mental health and wellbeing, with an aim to identify how schools might contribute to environments or ecologies of resilience. We investigated how access to resources for resilience is positively affected by factors such as youth identity, relationships, and belonging, which contributes to positive mental health, and conversely how psychological distress is increased by discrimination.

Positive wellbeing outcomes for youth from migrant backgrounds have been studied by Berry and colleagues [55], who noted that both maintenance of heritage culture and uptake of elements of the national culture of the settlement country are required for optimal wellbeing outcomes. Scholars such as Vacca et al. [78] and Tip et al. [79] further noted the importance of social networks that people from marginalized migrant backgrounds develop and extend, both within ethnic communities and with members of the broader population, which provide access to resources for wellbeing. Others have suggested that more complexified understandings of identity development, which take into account the ways in which heritage or ethnic identities are affected by discrimination, and how complex identities may act as protective mechanisms, are required [55,80]. Our findings with this cohort of Australian refugee background youth were not dissimilar to those of minority youth in Canada, where the impact of cultural identity on resilience was significant [81]. The positive influence of ethnic or cultural identity and belongingness has been noted elsewhere, for example, with immigrant adolescents in Germany [82]. These studies in Canada and Germany, in addition to the study presented here with refugee background youth in Australia, suggest that it is maintenance of connections to ethnic cultural heritage that are most crucial. In this study, not only was ethnic identity mediated by resilience to impact both life satisfaction and psychological distress, but it also had a direct effect on life satisfaction. These results indicate that participant youth can draw on relationships within cultural communities for emotional or practical support, that they felt a sense of belonging in the group, and that this contributed to overall resilience. We also note that our study suggested that although the impact of ethnic identity was most impactful (in the model), correlation analyses suggested a connection between having a strong sense of national identity and ethnic identity and positive mental health and wellbeing.

Ethnic identity was positively correlated with higher family congruence and a sense of belonging and support at school, indicating that the environment of resilience was not only impacted individually by these factors, but also that their interactions with each other were important. All of these factors significantly affected psychological distress and life satisfaction, as mediated by resilience. Considering Ungar et al.’s equifinality concept [33], these findings reinforce the notion that any improvements in one of these areas can positively impact resilience. Moreover, the correlations between exogenous factors suggest that improvements in one area are linked to overall improvements in all areas. In terms of school practice, these findings highlight how crucial a sense of belonging and support at school is for building a resilient environment, and how school support practices can be connected to whether youth feel a broader sense of belonging in their family or cultural community. These findings align with other research on belonging, which highlights the complexities of processes that contribute to identity development and a sense of belonging for young people from refugee backgrounds in resettlement contexts [83,84].

These three variables (ethnic identity, family congruence, and school support) were also negatively correlated with discrimination. Discrimination was tested for its negative impact on mental health outcomes, and mediation by resilience, with findings significant for a direct effect on psychological distress, a conclusion that persists in numerous studies with those from refugee backgrounds [18,51,85]. In this study, discrimination directly affected psychological distress for youth; however, the positive influence of other exogenous variables when mediated by resilience on both psychological distress and life satisfaction worked to ameliorate the negative influence of discrimination. In terms of school practice, these findings indicate two ways forward. Firstly, explicit anti-discrimination policies exist may need to be designed or redesigned, and additional practices may need to be explored that ensure effective implementation in schools [86]. Further research is needed to understand how these policies and practices can be designed and implemented effectively in light of the complex ways that students can experience discrimination by being othered in both subtle and explicit ways [87,88]. Secondly, that developing environments that contribute to resilience are key to improving wellbeing, even in the presence of discrimination.

The study investigated whether the gender or regional background of youth were significant factors affecting mental health. Although female participants reported lower resilience overall and lower life satisfaction, the positive influence of resilience on life satisfaction and psychological distress remained significant regardless of gender. Building resilient ecologies can therefore contribute to positive outcomes for both male and female youth. Similarly, although there was a slight variation in the significance of family congruence reducing psychological distress for males and females within regional groups, the overall impact of resilience on mental health and wellbeing remained significant. Experiences of discrimination were not significantly linked to either gender or region of origin, but were connected to time in Australia, indicating that anti-discrimination practices may wane as youth are considered more settled as opposed to being newcomers, or alternatively that young people become more attuned to racism as it manifests locally, although this requires further investigation in future research. The participant group in this study had a gender imbalance (one-third male, two-thirds female) and although this imbalance was accounted for in analyses, and significant results discussed, the gendered findings may require further study.

In terms of ways forward for school practice, and in addition to addressing discrimination as detailed above, connecting to ecologies of resilience outside of school, via family and cultural communities, could be an area for development. Resilient ecologies of youth in this study were linked, indicating that the interplay of these multiple ecologies is critical. School practice that incorporates a positive focus on different identities may contribute to resilience and positive mental health and wellbeing; although national identity was not a significant factor in the model it was earlier shown to significantly correlate to other elements of resilient ecologies including family congruence, ethnic identity, and mental health and wellbeing. Connections to families and communities that also contribute to resilience could improve mental health and wellbeing outcomes. Here it is also important to consider those for whom resilient ecologies were not present, who were disconnected from family, and from cultural community and identity, and felt unsupported at school, with low life satisfaction and increased psychological distress. School practices for these youth can take one of two approaches. One approach is to recognize when young people have poor mental health, such as increased psychological distress or reduced life satisfaction, and take that as an indicator to reach out to family and community to forge connections that contribute to ecologies of resilience. Alternatively, rather than waiting to identify mental health distress, which has been noted as challenging in cross-cultural settings within schools [89], school practice could incorporate connections to family and cultural communities and identity from the outset in order to build resilient ecologies that support mental health. In practice, both approaches may be necessary.

In the study, some assumptions regarding how best to understand issues relating to identity and belonging were required to collect and analyze quantitative data. Although the survey questions and scales were drawn from work in culturally diverse settings, it is an ongoing challenge for researchers that data of this kind can fail to adequately capture intersectional details or points of view [90]. Resilience research, in particular, can benefit from mixed-methods approaches [91]. It is an aim of this quantitative part of a wider mixed-methods study that some generalizable trends are noted that combine with deeper interrogation of qualitative data regarding the issues under investigation. The qualitative element of this mixed-methods study (published elsewhere) has revealed some of the ways in which the ecological systems that support resilience are navigated by participant youth. These findings offer insight into how youth navigate complex social networks, drawing on cultural wealth within families and ethnic or cultural communities [92], in addition to how broader ecological system dynamics impact experience [93].

This study involved youth in school and in contact with family. For youth disengaged from school or estranged from family, or separated through bereavement or geographical distance, support networks of different kinds may become more relevant. For students not in school, alternative pathways to employment and social connection may be crucial as they provide opportunity for other ecologies of resilience to be accessed and developed; this could be through vocational education pathways, employment pathways, or social groups such as faith-based communities or sport clubs [26]. These alternative pathways and the ways in which they contribute to resilient ecologies may be an avenue for further research.

## 5. Conclusions

Studies of resilience that focus on individual qualities or responses to circumstances can be prone to locating responsibility for resilience with the individual, drawing the focus away from the processes and mechanisms that contribute to environments or ecologies that support resilience, in this case for refugee background youth [89]. Research in this field is growing and evidence suggests that a focus on contextual environments and ecological systems that youth engage with is of key import [22].

The study showed how young people’s identity formation through, and sense of belonging within, families and school contexts were significant contributors to resilient ecologies. These intersecting ecologies promoted mental health and wellbeing for youth and ameliorated the negative impacts of discrimination. This focus on the contextual environment within which resilience is developed is crucial; resilience is due to multiple, nuanced, overlapping and complex systems that change over time [37], and it is by developing the ecologies of resilience that wellbeing can be supported. The study findings are relevant to development and implementation of school practices that support mental health and wellbeing.

The current findings suggest that school practice can contribute to resilience through connecting with and contributing to other ecologies of resilience, such as family and community networks, including cultural communities in addition to other communities, in order to provide multiple opportunities for youth to develop friendships, belonging, and connections. The findings also show that discrimination continues to have a negative effect on mental health and wellbeing, and so anti-discrimination practices are also needed to improve mental health and wellbeing outcomes for refugee background youth. Further study into the nature and extent of discrimination, and ways in which it can be reduced, is particularly salient given rising issues regarding cultural diversity and racialized exclusion in many countries that are also major humanitarian resettlement destinations [94].

## Figures and Tables

**Table 1:** Cronbach’s alpha for scales, means and standard deviations, and bivariate correlations.

	α	*M*	*SD*	1	2	3	4	5	6	7	8	9	10
1. Gender (M = 1, F = 2)	-	-	-	-									
2. Age (range 14–26 years)	-	17.57	2.32	0.10	-								
3. Time in Australia (range 1–15 years)	-	5.18	3.61	0.10	−0.05	-							
4. Discrimination (range 1–5)	0.80	2.27	0.92	−0.03	0.08	0.32 **	-						
5. Ethnic Identity (range 1–5)	0.89	4.10	0.82	0.04	−0.03	0.05	−0.20 **	-					
6. School Support (range 1–4)	0.86	3.26	0.59	0.03	−0.01	−0.19 **	−0.28 **	0.23 **	-				
7. Family Congruence (range 1–5)	0.90	3.94	0.85	0.05	−0.00	−0.13 *	−0.26**	0.34 **	0.27 **	-			
8. Life Satisfaction (range 1–5)	0.78	3.53	0.77	−0.14 *	−0.02	−0.06	−0.12 *	0.28 **	0.10	0.28 **	-		
9. Resilience (range 1–5)	0.86	3.60	0.71	−0.21 **	−0.07	0.08	−0.12 *	0.26 **	0.19 **	0.36 **	0.36 **	-	
10. Psychological Distress (range 10–50)	0.89	20.73	7.66	0.10	0.01	−0.10	0.20 **	−0.10	−0.09	−0.18 **	−0.27 **	−0.28 **	-
11. National Identity (range 1–5)	0.81	4.03	0.82	−0.10	0.04	−0.12 *	−0.24 **	0.33 **	0.31 **	0.35 **	0.15 **	0.19 **	−0.11

* Significant at the 0.05 level (2-tailed) ** Significant at the 0.01 level (2-tailed).

**Table 2:** Model fit.

	GFI	CFI	TLI	RMSEA	SRMR	χ2 (d*f*)	Δχ2(d*f*)	*p* (for χ2 or Δχ2)
Acceptable fit indices ^#^	≥0.9	≥0.95	≥0.9	≤0.08	≤0.09	Non-significant i.e., *p* > 0.05		
Model 1—Main path model	0.990	0.987	0.952	0.038	0.031	14.62(10)		*p* = 0.146
Model 2—Multigroup, regions (Africa/Middle East/South Asia) • Model 2A (unconstrained) • Model 2B (constrained weights) • Model 2C ^##^ (constrained covariances) • Model 2D ^##^ (constrained residuals)								
							
	0.972	0.965	0.874	0.036	0.048	42.72(30)	-	*p* = 0.062
	0.960	0.967	0.929	0.027	0.056	-	19.25(20)	*p* = 0.506
	0.922	0.911	0.896	0.033	0.073	-	62.25(42)	*p* = 0.023
	0.916	0.895	0.887	0.035	0.074	-	13.88(8)	*p* = 0.085
Model 3—Main path model with additional path from Family Congruence to Psychological Distress • Model 3A unconstrained model • Model 3B Multigroup (regions) (unconstrained)								
							
	0.991	0.985	0.942	0.042	0.030	14.018(9)	-	*p* = 0.122
	0.978	0.982	0.929	0.027	0.046	33.50(27)	-	*p* = 0.181

^#^ The comparative fit index (CFI), the Tucker–Lewis index (TLI), the root mean square error of approximation (RMSEA), the standardized root mean square residual (SRMR), chi-square (χ2) model fit indices drawn from Putnick and Bornstein [74], Hu and Bentler [75], Cheung and Rensvold [76], Fan, Thompson [77]. ^##^ Indication of possible invariance on some indices was considered acceptable within the context of acceptable alternate fit indices; multigroup analysis further explored these results.

**Table 3:** Standardized indirect effects (2000 bootstraps, 95%CI) mediated by resilience, direct effects, and total effects on two endogenous variables: significant effects displayed.

	Significant Indirect Effects			Significant Direct Effects			Total Effects		
	*β*	*p*	CI (95%)	*β*	*p*	CI (95%)	*β*	*p*	CI (95%)
**Psychological distress**									
Gender	0.060	0.001	0.026,0.108				0.060	0.001	0.026,0.108
Time in Australia	−0.040	0.001	−0.080, −0.015	−0.164	0.002	−0.258, −0.054	−0.204	0.002	−0.296, −0.091
Discrimination				0.215	0.001	0.096, 0.333	0.215	0.001	0.096,0.333
Ethnic Identity	−0.031	0.015	−0.068, −0.005				−0.031	0.015	−0.068, −0.005
Family Congruence	−0.078	0.001	−0.138, −0.038				−0.078	0.001	−0.138, −0.038
School Support	−0.028	0.028	−0.068, −0.003				−0.028	0.028	−0.068, −0.003
**Life Satisfaction**									
Gender	−0.075	0.001	−0.127, −0.036				−0.075	0.001	−0.127, −0.036
Time in Australia	0.050	0.001	0.020, 0.089				0.050	0.001	0.020, 0.089
Ethnic Identity	0.038	0.017	0.006, 0.083	0.200	0.001	0.083, 0.319	0.239	0.001	0.113, 0.356
Family Congruence	0.097	0.001	0.048, 0.164				0.097	0.001	0.048, 0.164
School Support	0.034	0.032	0.003, 0.080				0.034	0.032	0.003, 0.080

## Data Availability

The data from this study are not publicly available due to confidentiality concerns.
